# Opinions, Views, and Expectations Concerning the Radiology Report: A Rural Medicine Report

**DOI:** 10.7759/cureus.5822

**Published:** 2019-10-02

**Authors:** Tej I Mehta, Aikaterina Assimacopoulos, Caleb J Heiberger, Simcha Weissman, Douglas Yim

**Affiliations:** 1 Radiology, University of South Dakota Sanford School of Medicine, Sioux Falls, USA; 2 Obstetrics and Gynecology, University of South Dakota Sanford School of Medicine, Sioux Falls, USA; 3 Internal Medicine, Hackensack University Medical Center, North Bergen, USA; 4 Interventional Radiology, Avera McKennan Hospital and University Health Center, Sioux Falls, USA

**Keywords:** quality improvement, radiology, report, communication, education, interdisciplinary education

## Abstract

This study seeks to examine a potential agreement and/or discordance of specific aspects of the radiology report between referring clinicians and radiologists within a medical group in a predominately rural setting. This study also aims to compare results with similar studies conducted in other geographic regions. This was done using a previously validated survey tool that examines five different aspects of the radiology report: importance, clinical correlation, clinicians’ satisfaction, content, structure, and style. Dichotomized results were statistically analyzed using *χ*^2^ or Fischer’s exact test and showed significant differences in the areas of importance and content. Non-dichotomized results unique to clinicians and radiologists were assessed qualitatively. Most clinicians found the radiology report to be useful in their clinical decision making and that they received radiology reports in a timely enough fashion to affect their decision making. These results were largely found to be in accordance with similar studies, but significant differences unique to the sampled population were present. Based on these findings, we have included specific recommendations that may enhance the clinical efficiency of radiology reports as used by clinicians and potentially reduce medical errors secondary to clinical information not always fully captured in radiology reports.

## Introduction

Effective communication between radiologists and clinicians is important for patient management. The American College of Radiology (ACR) provides three major guidelines for effective communication of diagnostic imaging findings including meeting the need for timeliness of reports, encouraging physician-to-physician communication, and minimizing the risk of communication errors [1]. Therefore, enhanced provider communication is a fundamental aspect of improved patient care.

For roughly 40 years, studies have been published regarding the efficacy, efficiency, and clinical utility of the radiology report [2]. Studies have consistently indicated that referring clinicians thought the radiology report to be indispensable to medical practice, have preferred that radiologists include more detail in their reports, and have indicated that they prefer itemized reporting [2]. Moreover, nearly all major studies concerning the radiology report have highlighted the importance of radiologists learning to report well and that reporting be a focus of future training for radiologists, yet most centers in the US do not provide more than one hour per year of radiology reporting education [3].

Previous studies have investigated the opinions and expectations concerning the radiology report to identify areas for quality improvement and to increase communication between referring clinicians and radiologists. These studies have taken place in a variety of geographic regions including the European Union (EU) countries and the Philippines [2,4]. However, these studies have been mostly qualitative in nature and have indicated that providers in specific geographic regions may have different preferences regarding radiology reporting. The study detailed herein was designed in a similar nature to those previously conducted, but with added quantitative survey sections to better characterize the opinions and expectations concerning radiology reports. Moreover, this study was conducted in a primarily rural state with input from providers in small to medium-sized communities.

This article reports the results of two cross-sectional surveys regarding the opinions, views, and expectations concerning the radiology report conducted within a medical group in the state of South Dakota. The purpose of this study was to both qualitatively and quantitatively compare opinions and expectations concerning the radiology report between clinicians and radiologists in a midwestern, primarily rural setting and the further compare these data to previously published studies of a similar cross-sectional design.

## Materials and methods

Two surveys were generated for this study: Clinicians’ Opinions, Views, and Expectations concerning the radiology Report (COVER) and Radiologists’ Opinions, Views and Expectations concerning the radiology Report (ROVER). Each survey contained three sections: one section concerning demographic information, one concerning quantitative information pertaining to factors such as timeliness of radiology reporting, and another section concerning qualitative information regarding the radiology report, such as language and style. For the quantitative and qualitative questions, respondents were asked to indicate their level of agreement on a modified, four-tiered Likert scale. Quantitative questions were tiered “100% of the time - 76% of the time”, “75% of the time - 51% of the time”, “50% of the time - 26% of the time” and “25% of the time - 0% of the time”. Qualitative questions were tiered “Mostly agree”, “Somewhat agree”, “Somewhat disagree”, and “Mostly disagree”. COVER consisted of 28 questions and ROVER consisted of 27 questions. Twenty questions were pair-matched between the surveys for comparison with eight and seven questions, respectively, concerning background information of interest (including demographic questions). Pair-matched questions were grouped by the following categories: Importance of the Radiology Report, Satisfaction with the Radiology Report, Clinical Correlation, Content of the Radiology Report, and Structure/Style of the Radiology Report.

Survey creation and data collection were conducted using Google Forms. Clinicians and radiologists were invited to participate via email if they were identified as a practicing physician within our medical group. Surveys remained open for one month following the distribution of emails. All data were collected over a one-month period in the fall of 2018. The data were transferred to Microsoft Excel files and imported into R for statistical analysis. The overall results of the tiered modified Likert scale were complemented by columns in which the top two and the bottom two categories (i.e. “100% of the time - 76% of the time” and “75% of the time - 51% of the time”) were combined into total categories of “≥51% of the time” and “<50% of the time” or “Agree” and “Disagree” to indicate the net skewness of responses. Clinician and radiologist responses were compared using the chi-square test for independence or Fischer's exact test if normality could not be assumed. *P*-values of <0.05 were considered to indicate a statistically significant difference. Permission to conduct this study was obtained from our institutional review board.

## Results

All results were screened for completeness. In the event, responders skipped one or more statements; only the statements that had been ranked were retained for the study. In total, 103 clinicians and seven radiologists responded to the surveys (12% and 20% response rates, respectively). Sex and age distribution were in approximate accordance with the underlying sex and age distributions of practicing physicians at this institution. Data are presented as paired tables for clinicians and radiologists with “C:” signifying a clinician response and “R:” signifying a radiologist response. *P*-values for analyses on the difference in response distributions between clinicians and radiologists are presented on the respective lines for radiologists only to reduce redundancy.

Clinician and radiologist demographics

Clinician and radiologist demographics fit the expected distribution of our medical group physician demographic profile. Approximately 85.4% of clinician respondents and 85.7% of radiologist respondents were males (Table 1). Clinician age groups fit an approximately normal distribution, with approximately 50% of respondents within the 36 to 54 years of age category, and 71.4% of radiologist respondents fit the same age category (Table 1). There was a large difference in the race/ethnicity of clinician and radiologist respondents with 86.4% of clinician respondents and 57.1% of radiologist respondents identifying as “non-Hispanic White” (Table 1). However, this is in keeping with the demographic profile of this institution. Approximately 73.8% of clinician respondents primarily work in an outpatient setting. The majority of clinician respondents were primary care providers. The plurality of specialty responses was from family medicine/general practice, internal medicine, and pediatrics (Table 1).

**Table 1:** Demographics of clinicians and radiologists

Clinicians		Radiologists	
Gender	No. (%)	Gender	No. (%)
Male	88 (85.4%)	Male	6 (85.7%)
Female	15 (14.6%)	Female	1 (14.3%)
Age (years)	No. (%)	Age (years)	No. (%)
24-35	27 (26.2%)	24-35	1 (14.3)
36-54	52 (50.5%)	36-54	5 (71.4)
55-70	24 (23.3%)	55-70	1 (14.3)
Race/Ethnicity	No. (%)	Race/Ethnicity	No. (%)
Middle Eastern or Arab	2 (1.9)	East Asian	1 (14.3)
Non-Hispanic White	89 (86.4)	Non-Hispanic White	4 (57.1)
South Asian	8 (7.8)	South Asian	2 (28.6)
Southeast Asian	4 (3.9)		
Primary Work Environment	No. (%)		
Inpatient setting	27 (26.2)		
Outpatient setting	76 (73.8)		
Specialty/Subspecialty	No.		
Anaesthesiology	5		
Emergency medicine	4		
Endocrinology	1		
Family Medicine/General practitioner	21		
Gastroenterology	2		
General Surgery	5		
Internal Medicine	24		
Neurology	1		
Obstetrics and Gynecology	1		
Orthopedics	1		
Other	5		
Pediatrics	23		
Pediatrics, Sport medicine	1		
Prefer not to respond	6		
Pulmonology	1		
Radiation Oncology	1		
Urology	1		

The importance of the radiology report

On the question of radiologist competency at image interpretation, 83% of clinicians and 100% of radiologists felt that radiologists are more competent than clinicians at least 51% of the time (*p* = 0.50). Moreover, 64% of clinicians and 100% of radiologists felt this to be true at least 76% of the time (Table 2).

**Table 2:** Importance of the radiology report

Question/Statement	100% to 76% of the time	75% to 51% of the time	50% to 26% of the time	25% to 0% of the time	Result	p-value
C: Radiologists overall have greater competence at radiographic interpretation than clinicians	66	19	8	10	≥51% of the time	
R: Radiologists overall have greater competence at radiographic interpretation than clinicians	7	0	0	0	≥51% of the time	0.5
C: Radiology reports mention important issues clinicians may not notice	12	24	29	38	≤50% of the time	
R: Radiology reports mention important issues clinicians may not notice	2	3	1	1	≥51% of the time	0.05
C: Clinicians read at least some of the radiology report	76	19	0	8	≥51% of the time	
R: Clinicians read at least some of the radiology report	2	3	1	1	≥51% of the time	0.06
C: Clinicians only read the impression/conclusion of a radiology report	5	10	46	41	≤50% of the time	
R: Clinicians only read the impression/conclusion of a radiology report	3	3	1	0	≥51% of the time	<0.01
C: Clinicians read the entirety of the radiology report	33	35	15	20	≥51% of the time	
R: Clinicians read the entirety of the radiology report	1	1	1	4	≤50% of the time	0.02

Thirty-five percent of clinicians and 71% of radiologists indicated that the radiology report mentions important issues clinicians may not notice at least 51% of the time (*p* = 0.05), indicating net disagreement between radiologists and clinicians on this question at the level of significance (Table 2).

Ninety-two percent of the clinicians indicated that they read at least some of the radiology reports 51% of the time or more; 71% of radiologists indicated that they believed clinicians read at least some of the radiology report 51% of the time or more (Table 2). Fifteen percent of the clinicians indicated that they only read the impression/conclusion of the radiology report 51% of the time or more, whereas 86% of the radiologists indicated that they believe most clinicians only read the impression/conclusion 51% of the time or more (*p *< 0.01). In addition, 66% of clinicians indicated that they read the entirety of the radiology report more than 51% of the time; however, only 29% of radiologists indicated that they believed clinicians read the radiology report more than 51% of the time (*p* = 0.02; Table 2).

Satisfaction with the radiology report

Overall, there were no significant differences between clinicians and radiologists regarding overall satisfaction with the radiology report. Only 5% of clinicians and no radiologists indicated that the radiology report is difficult to understand due to language/style issues more than 51% of the time (*p *= 1.00). Moreover, only 13% of clinicians and 14% of radiologists indicated that the radiology report is difficult to understand due to language/style issues more than 25% of the time, while 95% of clinicians and 86% of radiologists indicated that radiology reports are adequately proofread 51% of the time or more (*p* = 0.28; Table 3).

**Table 3:** Quantitative satisfaction with the radiology report

Question/Statement	100% to 76% of the time	75% to 51% of the time	50% to 26% of the time	25% to 0% of the time	Result	p-value
C: There are language/style issues in radiology reports that make them more difficult to understand	1	4	9	89	≤50% of the time	
R: There are language/style issues in radiology reports that make them more difficult to understand	0	0	1	6	≤50% of the time	1.00
C: Radiology reports are proofread before they are sent	89	9	5	0	≥51% of the time	
R: Radiology reports are proofread before they are sent	4	2	0	1	≥51% of the time	0.28

Most clinicians (86%) and 100% of radiologists agreed with the statement “Radiology reports are easily understood” (*p *= 0.60). Approximately 73% of clinicians and 100% of radiologists disagreed with the statement “If a radiology report is not understood, the fault lies with the radiologists’ phrasing, not the clinicians’ interpretation” (*p *= 0.19; Table 4).

**Table 4:** Qualitative satisfaction with the radiology report

Question/Statement	Mostly agree	Somewhat agree	Somewhat disagree	Mostly disagree	Result	p-value
C: Radiology reports are easily understood	64	25	12	1	Agree	
R: Radiology reports are easily understood	5	2	0	0	Agree	0.60
C: If a radiology report is not understood, the fault lies with the radiologists phrasing, not the clinicians' interpretation	3	25	55	20	Disagree	
R: If a radiology report is not understood, the fault lies with the radiologists phrasing, not the clinicians' interpretation	0	0	4	3	Disagree	0.19

Clinical correlation

There were no significant differences regarding the clinical correlation of radiology reports. Clinicians and radiologists both overwhelmingly agreed with the statements “To make a good report, the radiologist has to know the medical condition of the patient” (97% and 100% respectively, *p *= 1.00) and “To make a good report, the radiologist has to know what the clinical question is” (100% and 100%, respectively, *p *= 1.00; Table 5).

**Table 5:** Clinical correlation of the radiology report

Question/Statement	Mostly agree	Somewhat agree	Somewhat disagree	Mostly disagree	Result	p-value
C: To make a good report, the radiologist has to know the medical condition of the patient	55	45	3	0	Agree	
R: To make a good report, the radiologist has to know the medical condition of the patient	4	3	0	0	Agree	1.00
C: To make a good report, the radiologist has to know what the clinical question is	84	19	0	0	Agree	
R: To make a good report, the radiologist has to know what the clinical question is	5	2	0	0	Agree	1.00

Content of the radiology report

The only significant difference between clinicians and radiologists concerning the content of the radiology report was regarding the question “The descriptive part of a report contains influential information not otherwise contained within the impression of the report.” Seventy-eight percent of the clinicians and 43% of radiologists agreed with this statement (*p* = 0.04; Table 6). There was a net agreement between the clinicians and radiologists regarding the questions “When a simple examination (e.g., a chest X-ray) does not show anything abnormal, the report impression can be limited to a mere: “Normal chest x-ray”” and “When a complex examination (e.g., an ultrasonography of the abdomen) does not show anything abnormal, the report impression can be limited to a mere: “Normal ultrasound of the abdomen” (Table 6).

**Table 6:** Content of the radiology report

Question/Statement	Mostly agree	Somewhat agree	Somewhat agree	Mostly disagree	Result	p-value
C: When a simple examination (eg, a chest X-ray) does not show anything abnormal, the report impression can be limited to a mere: “Normal chest x-ray”	69	4	30	0	Agree	
R: When a simple examination (eg, a chest X-ray) does not show anything abnormal, the report impression can be limited to a mere: “Normal chest x-ray”	5	1	0	1	Agree	0.40
C: When a complex examination (e.g., ultrasonography of the abdomen) does not show anything abnormal, the report impression can be limited to a mere: “Normal ultrasound of the abdomen”	45	20	20	18	Agree	
R: When a complex examination (e.g., ultrasonography of the abdomen) does not show anything abnormal, the report impression can be limited to a mere: “Normal ultrasound of the abdomen”	1	4	0	2	Agree	0.66
C: The descriptive part of a report contains influential information not otherwise contained within the impression of the report	25	55	15	8	Agree	
R: The descriptive part of a report contains influential information not otherwise contained within the impression of the report	3	0	1	3	Disagree	0.04
C: Not mentioning a particular organ or body part in a radiology report implies the radiologist has not looked at it closely	30	35	35	3	Agree	
R: Not mentioning a particular organ or body part in a radiology report implies the radiologist has not looked at it closely	1	2	1	3	Disagree	0.29
C: If the report is short, the radiologist has not looked at the image(s) thoroughly	1	21	51	30	Disagree	
R: If the report is short, the radiologist has not looked at the image(s) thoroughly	0	3	1	3	Disagree	0.19

There was a net difference between clinicians and radiologists regarding the question “Not mentioning a particular organ or body part in a radiology report implies the radiologist has not looked at it closely” with 63% of clinicians and 43% of radiologists agreeing with this statement; however, the difference did not reach the level of significance (*p* = 0.29). Both clinicians and radiologists disagreed with the statement “If the report is short, the radiologist has not looked at the image(s) thoroughly” (79% and 57% respectively, *p* = 0.19).

Structure and style of the radiology report

There were no significant differences regarding the structure and style of the radiology report. Fifty-eight percent of the clinicians and 43% of the radiologists agreed with the statement “Radiology reports should end with a recommendation/plan” (*p *= 0.43). Sixty-three percent of the clinicians and 57% of the radiologists agreed with the statement “A report should consist of a fixed list of short descriptions of the findings (as opposed to prose)” (*p *= 0.75). Eighty-two percent of the clinicians and 100% of the radiologists agreed with the statement “Radiology reports should be divided based on individual organ systems” (*p *= 0.60). 78% of clinicians and 100% of radiologists agreed with the statement “The simpler the style and vocabulary of a radiology report, the better the message will be understood” (*p* = 0.34; Table 7).

**Table 7:** Structure and style of the radiology report

Question/Statement	Mostly agree	Somewhat agree	Somewhat disagree	Mostly disagree	Result	p-value
C: Radiology reports should end with a recommendation/plan	15	45	25	18	Agree	
R: Radiology reports should end with a recommendation/plan	2	1	3	1	Disagree	0.43
C: A report should consist of a fixed list of short descriptions of the findings (as opposed to prose)	25	40	35	3	Agree	
R: A report should consist of a fixed list of short descriptions of the findings (as opposed to prose)	3	1	2	1	Agree	0.75
C: Radiology reports should be divided based on individual organ systems	49	35	15	4	Agree	
R: Radiology reports should be divided based on individual organ systems	4	3	0	0	Agree	0.60
C: The simpler the style and vocabulary of a radiology report, the better the message will be understood	55	25	19	4	Agree	
R: The simpler the style and vocabulary of a radiology report, the better the message will be understood	6	1	0	0	Agree	0.34

Unpaired questions

Sixty-nine percent of clinicians reported that they read the radiology report as soon as it is available 51% of the time or more. Approximately 85.4% of the clinicians reported that they receive radiology reports in a timely enough fashion to affect their medical decisions 51% of the time or more, while 55.3% of clinicians noted this to occur 76% of the time or more for the same question. Approximately 91.2% of clinicians agreed with the statement “radiology reports are useful in my medical decision making” (Table 8).

**Table 8:** Unpaired clinician questions

Question/Statement	100% to 76% of the time	75% to 51% of the time	50% to 26% of the time	25% to 0% of the time	Result
I read a radiology report as soon as it is available	52 (50.5)	19 (18.4)	24 (23.3)	8 (7.8)	≥51% of the time
I receive radiology reports in a timely enough fashion to affect my clinical decisions	57 (55.3)	31 (30.1)	10 (9.7)	4 (3.9)	≥51% of the time
Radiology reports are useful in my medical decision making	64 (62.1)	30 (29.1)	8 (7.8)	1 (1.0)	≥51% of the time

Seventy-one percent of radiologists indicated that they “provide more than one differential in my impression of a radiology report” 51% of the time or more (Table 9).

**Table 9:** Quantitative unpaired radiologist questions

Question/Statement	100% to 76% of the time	75% to 51% of the time	50% to 26% of the time	25% to 0% of the time	Result
I provide more than one differential in my impression of a radiology report	3 (42.9)	2 (28.6)	1 (14.3)	1 (14.3)	≥51% of the time

One-hundred percent of the radiologists agreed with the statement “Not taking into account my competence as a radiologist, my reports are better written than my colleagues’”. Approximately 85.7% of radiologists agreed with the statement “I improve my radiology reports by reading the reports of my colleagues”. Approximately 28.6% of radiologists agreed with the statement “I spend a disproportionate amount of my day writing radiology reports as opposed to interpreting radiologic studies” (Table 10).

**Table 10:** Qualitative unpaired radiologist questions

Question/Statement	Mostly agree	Somewhat agree	Somewhat agree	Mostly disagree	Result
Not taking into account my competence as a radiologist, my reports are better written than my colleagues’	3 (42.9)	4 (57.1)	0 (0.0)	0 (0.0)	Agree
I improve my radiology reports by reading the reports of my colleagues	4 (57.1)	2 (28.6)	1 (14.3)	0 (0.0)	Agree
I spend a disproportionate amount of my day writing radiology reports as opposed to interpreting radiologic studies	1 (14.3)	1 (14.3)	2 (28.6)	3 (42.9)	Disagree

## Discussion

The radiology report tells a story that must honestly represent the available information including both the images and the clinical status of the patient. However, that information must be synthesized and concentrated in such a way that the radiologist is able to provide a summary of the images, highlight the salient details and ultimately craft something of clinical utility. While the radiologists' skills at image interpretation and diagnosis are highly emphasized in training, the skills of synthesizing, concentrating and communicating the radiologists’ interpretation are not highly emphasized in training [3,5-6]. In this process, certain biases are likely at play and the expectations of clinicians and radiologists may not always be in line. As such, the goal of this study was to highlight areas of agreement and disagreement concerning the radiology report between clinicians and radiologists.

On the topic of importance, radiologists and clinicians agreed on the competence of radiologists but disagreed about aspects of clinical correlation. Radiologists overall believed that the radiology report mentions important issues a clinician may otherwise not notice at least 51% of the time, whereas clinicians overall believed this to be less than 51% of the time. The other major COVER style studies, Southeast Asian (SEA) and European (EURO), reported 79.4% and 58.9% of the clinicians agreeing with the radiologists for the equivalent question [2,4]. The differences identified here raise the question of what do radiologists and clinicians deem to be important clinical issues? There is little information on this question in the literature; however, Wallis and McCourbrie highlighted the importance of appropriately conveying radiological findings and answering clinical questions [7]. In a prospective study conducted by Wallis et al., 61.7% of assessors independently agreed to the question “Does the (radiology) report add clinical value to patient management?” [8]. Further investigations into this topic are warranted to better assess the clinical value of radiology reports. We would postulate that specific specialties, such as surgical specialties, may overall find greater clinical utility from radiology reports than other specialties. Moreover, it is notable that different geographic regions appear to have large differences of opinion regarding the clinical importance of radiology reports. Radiologists and clinicians disagreed about how much of and how often clinicians read the entirety of the report with radiologists believing clinicians read the entire report 50% of the time or less and clinicians reporting they read the entire report 51% of the time or more. There was no equivalent statement in either the SEA or EURO studies.

On the topic of the content of the radiology report, there was significant disagreement on the statement "The descriptive part of a report contains influential information not otherwise contained within the impression of the report". Seventy-eight percent of the clinicians and 43% of the radiologists agreed with this statement. This result implies that clinicians believe information pertinent to their medical decisions is not fully captured within the impression and they may have to go “hunting” for information elsewhere in the report. A majority of respondents in the SEA and EURO studies agreed with the statement “The descriptive part of the report should also be read, not only the conclusion”; this may imply clinicians believe reading the descriptive section simply gives them a better understanding of the patient’s clinical picture or that the impression of a report truly does not sufficiently capture the relevant clinical information [2,4]. In another cross-sectional survey, McLoughlin et al. found that the extent of detail in the descriptive section of radiology reports desired by clinicians was dependent on clinical circumstances [9].

We did not find any statistically significant differences between clinicians and radiologists regarding satisfaction with radiology reports, the importance of clinical correlation nor on structure and style. Reports appear to be well-understood by clinicians, without language/style issues, and with adequate proofreading prior to being sent. Agreement on the questions of clinical correlation is a hopeful and positive outcome. The older norm among many radiologists of ignoring the patient’s clinical status or of clinicians omitting clinical information to complement image interpretation does not seem to exist in any significant way at our institution. This is encouraging because several studies have indicated that reliable clinical information and discreet examination questions from the referring clinician can significantly improve diagnostic accuracy [7,10-11]. These results are in agreement with the SEA and EURO COVER studies.

Limitations

This study posed several limitations. The first is a potential variability in the respondent’s interpretation of the questions. Another is the presence of recall bias whereby clinicians and radiologists are asked to report the average frequency of past events over long periods of clinical practice. The Hawthorne effect may be present in that clinician’s responses to questions may reflect on their habits of practice and therefore have the potential to be inflated such that their responses are more aligned with what they believe to be best practice rather than what may occur [12]. The study was also limited by a small sample size which was accounted for, in part, using non-parametric statistical analysis. Unfortunately, while this survey-based study had a good radiologist response rate in terms of percent of practicing radiologists in our group, the absolute number of radiologists was small, thus reducing the statistical power of the study. Finally, the study does not allow for follow-up questions of participating respondents due to the anonymous nature of the survey.

One limitation noted in previous studies was that in asking participates to respond by expressing "degree of agreement" the questions asked only for qualitative information and allowed for a wider degree of interpretation [2,4]. An attempt was made to account for this and provide more quantitative data with the modified Likert scale whereby respondents were asked about the frequency of occurrence where applicable rather than the degree of agreement. We feel that for select questions a modified Likert approach indicating the perceived frequency of events may provide more clinically applicable information for select clinical topics.

## Conclusions

The results of this study serve as a guide to encourage clinician and radiologist communications and clarify expectations regarding the radiology reports. At our institution, aspects of structure and style showed the greatest potential for improving communication and utility of radiology reports among clinicians. Over the last decade, efforts have been made to establish structured reporting practices with an emphasis on robustness and clinical utility. Furthermore, a greater emphasis on quality measures for diagnostic imaging by radiographic societies has likely helped enhance communication between radiologists and clinicians. More studies of a similar nature to the one detailed herein in various geographic locales could provide a better picture of opinions, views, and expectations concerning the radiology report and more time spent in radiology residency on communication could increase the clinical utility of reports.
